# Supplementary material for: Characterisation of meteorological drought at sub-catchment scale in Afghanistan using station-observed climate data
Source: PLoS One. 2023 Feb 6;18(2):e0280522. doi: 10.1371/journal.pone.0280522 (PMC9901756; doi:10.1371/journal.pone.0280522)
Supplement: Attachment — Submitted filename: Characterization of meteorological drought at sub-catchment scale in Afghanistan using station-observed climate data.pdf [file pone.0280522.s004.pdf]

Reviewers' comments:

The manuscript entitled " **Characterization of meteorological drought at sub-catchment scale in Afghanistan using station-observed climate data**" assessed the spatiotemporal of drought characteristic (distribution, frequency, severity and duration) over different sub-catchment of Afghanistan using Standardized Precipitation Evapotranspiration Index (SPEI) at different time scales. The paper is written well and investigated an important topic related to Afghanistan where such studies are very rare. I have some minor comments that can be useful to authors.

Abstract: "Droughts have severely affected Afghanistan over the last two decades" in introduction section line 2 its written over the last four decades instead of two decades, please check which one is correct.

Keywords: Please include Afghanistan as a keyword.

Introduction:

P1: "The 2018 drought, which was one of the most severe droughts in Afghanistan in the last four decades" in abstract part the most recent drought is written as 2011. Please check it.

P13: "58% of Afghanistan, mainly located in the south of the cou. This dryness" please correct the word "cou".

P18 "Figure 11. Long-term annual maximum drought duration at sub-catchment scale in Afghanistan (1980-2017) based on SPEI-3." Please check figure 11 caption is long-term drought duration or spatial distribution of drought severity?
