# Supplementary material for: Characterisation of meteorological drought at sub-catchment scale in Afghanistan using station-observed climate data
Source: PLoS One. 2023 Feb 6;18(2):e0280522. doi: 10.1371/journal.pone.0280522 (PMC9901756; doi:10.1371/journal.pone.0280522)
Supplement: Attachment — Submitted filename: Response to Reviewers.docx [file pone.0280522.s005.docx]

**Response to Reviewers**

Reviewer #1

**General Comment**

The manuscript entitled " Characterization of meteorological drought at sub-catchment scale in Afghanistan using station-observed climate data” assessed the spatiotemporal of

drought characteristic (distribution, frequency, severity and duration) over different subcatchment of Afghanistan using Standardized Precipitation Evapotranspiration Index (SPEI)

at different time scales. The paper is written well and investigated an important topic

related to Afghanistan where such studies are very rare. I have some minor comments that

can be useful to authors.

**Specific Comments**

***Comment****: Abstract: “Droughts have severely affected Afghanistan over the last two decades” in*

*introduction section line 2 its written over the last four decades instead of two decades,*

*please check which one is correct.*

**Response**: Thanks for pointing out this. Revised to “four decades”.

***Comment****: Keywords: Please include Afghanistan as a keyword.*

**Response**: Added.

***Comment****: Introduction: P1: “The 2018 drought, which was one of the most severe droughts in Afghanistan in the last four decades” in abstract part the most recent drought is written as 2011. Please check it.*

**Response**: “four decades” is correct. Have revised the first sentence in Abstract accordingly.

***Comment****: P13: “58% of Afghanistan, mainly located in the south of the cou. This dryness” please*

*correct the word “cou”.*

**Response**: Done.

***Comment****: P18 “Figure 11. Long-term annual maximum drought duration at sub-catchment scale in*

*Afghanistan (1980-2017) based on SPEI-3.” Please check figure 11 caption is long-term*

*drought duration or spatial distribution of drought severity?*

**Response**: The caption of Figure 11 is correct. It shows the annual max drought duration base on SPEI-3.

Reviewer #2

**General Comment**

The manuscript “Characterisation of meteorological drought at sub-catchment scale in Afghanistan using station-observed climate data” presents an interesting research which is of importance to the area of study. I have the following comments and suggestions for the authors.

**Specific Comments**

**ABSTRACT**
***Comment****: I suggest the authors add some numerical results such as some significant SPEI values obtained in the study area.*
**Response**: A couple of sentences of the key drought characteristics representing some significant SPEI values have been added into Abstract.

**INTRODUCTION**

***Comment****: Paragraph 1: Authors should highlight other drought occurences and their impacts in Afghanistan.*

**Response**: Yes, more droughts and their impact have been highlighted.

***Comment****: Paragraph 4: remove dimensional from the sentence.*
**Response**: Removed.

***Comment****: In the second to the last paragraph, the authors discussed the constraints faced by the previous studies to include use of use of out-of-date long-term historical data; use of gridded global datasets at 0.5o (or ~ 50km) resolutions. Can the authors justify how these are constraints especially since some studies have used data of such resolutions and others have found gridded data sets to be able to replicate observed data sets. The following studies are example:*

- *Changing characteristics of meteorological droughts in Nigeria during 1901–2010,* [*https://doi.org/10.1016/j.atmosres.2019.03.010*](https://doi.org/10.1016/j.atmosres.2019.03.010)
- *Comparison of precipitation projections of CMIP5 and CMIP6 global climate models over Yulin, China,* [*https://doi.org/10.1007/s00704-021-03823-6*](https://doi.org/10.1007/s00704-021-03823-6)

**Response**: This is a very good comment. We understand your point. We have no objections to the use of gridded global dataset at relatively coarse resolutions as along as they are properly validated using enough ground stations. Based on our experience of long-term working in the south and central Asia, the low density of ground stations in some regions used in some global datasets resulted in poor interpolation results. Therefore, the accuracy of a global dataset could vary over different areas. In this study, we were also able access to the ‘best available’ station data collected by Afghanistan’s water monitoring agency. The reasonable station data is unlikely to be accessed in other Afghanistan studies. However, we appreciate the reviewer’s concern. We have slightly changed the relevant sentences to make the more specifically to Afghanistan. We also cited one of the above references in Introduction.

**SUTDY AREA AND DATA**
**Description of study area**
***Comment****:* Remove ‘at’ from “Afghanistan is located at in southern-central Asia (Figure 1).”
**Response**: Removed.

*Comment: Figure 1 shows there are no existing gauge stations in most of the south of the study area, which may result into some catchments not having any stations within them. How were the authors able to capture the precipitation characteristics of the areas especially since the authors consider interpolation and use of gridded data sets as limitations in the introductory section?*
**Response**: This is a good question. In this study, we have used some cross border stations in Pakistan to assist with the necessary interpolation which capture the precipitation characteristics of the areas to minimise errors and uncertainties.

***Comment****: I suggest some part of section 2.2.3 like the Hargreaves and Samani method especially the equations described be moved to the methodology section as they are more of method than data.*
**Response**: Part of 2.2.3 for calculating PET has been moved to the Methodology section as 3.1.

***Comment****: Also, why did the authors choose the Hargreaves and Samani method of estimating the PET since the Penman-Monthieth method is always recommended for SPEI calculation and since there can be significant differences amongst the SPEI series estimation by the different PET methods in some regions; like larger in semi-arid to mesic regions and smaller in humid regions.*
**Response**: The reviewer is correct. The authors would have used Penman-Monteith if we had the necessary station data (i.e. radiation, soil heat flux, air density, wind speed). In the paper, we have added a sentence explaining the limitation: “An improvement on the calculation of PET would be to use Penman-Monteith, which is always recommended for SPEI calculation. There can be significant differences amongst the SPEI series estimation by the different PET methods in some regions; like larger values in semi-arid to mesic regions and smaller values in humid regions. Unfortunately, station data was not available to apply Penman-Monteith in this study.”

**DISCUSSION
*Comment****: How has the authors tried to reduce the possible uncertainties reported in this section?*
**Response**: Since this study is based observed data, data quality is the major driver to reduce the possible uncertainties reported in this section. We were able to access to ‘best available’ station data collected by Afghanistan’s water monitoring agency through a strict data QA process. We also paid special attention to the section of gap-filling approach for missing data and interpolation method for no-station sub-catchments if needed.

***Comment****: It would be interesting if the authors could add a paragraph or two discussing the droughts in the country has affected agricultural output in the region.*

**Response**: Thanks for your suggestion. The impact of meteorological drought on agricultural output can be best estimated using a series of agricultural drought indices (including surface runoff, soil moisture and vegetation data). Without a such a quantitative measure being undertaken by ourselves, we feel we are not in a strong position to discuss this topic (given the scope of this paper is characterising meteorological drought in Afghanistan). In addition, estimated agriculture losses resulting from drought can be found from the FAO report (2019) in the list of references. The good news is we are in the process of analysing vegetation response to drought in Afghanistan using drought indices derived from climate data and vegetation indices derived from remotely sensed images (1980-present). This will lead to another research paper contributing to agricultural drought assessment.
